# The evidence of human exposure to glyphosate: a review

**DOI:** 10.1186/s12940-018-0435-5

**Published:** 2019-01-07

**Authors:** Christina Gillezeau, Maaike van Gerwen, Rachel M. Shaffer, Iemaan Rana, Luoping Zhang, Lianne Sheppard, Emanuela Taioli

**Affiliations:** 1 Institute for Translational Epidemiology and Department of Population Health Science and Policy, Icahn School of Medicine at Mount Sinai, One Gustave L. Levy Place, Box 1133, New York, NY 10029 USA; 2 Department of Environmental and Occupational Health Sciences, University of Washington, 1959 NE Pacific St, Seattle, WA 98195 USA; 3 Division of Environmental Health Sciences, School of Public Health, University of California Berkeley, 2121 Berkeley Way, Room 5302, Berkeley, CA 94720-7360 USA; 4 Department of Biostatistics, University of Washington, Box 357232, Seattle, WA 98195-7232 USA

**Keywords:** Glyphosate, Round-up, Herbicides, Environmental carcinogens, Human biomonitoring, Exposure assessment

## Abstract

**Background:**

Despite the growing and widespread use of glyphosate, a broad-spectrum herbicide and desiccant, very few studies have evaluated the extent and amount of human exposure.

**Objective:**

We review documented levels of human exposure among workers in occupational settings and the general population.

**Methods:**

We conducted a review of scientific publications on glyphosate levels in humans; 19 studies were identified, of which five investigated occupational exposure to glyphosate, 11 documented the exposure in general populations, and three reported on both.

**Results:**

Eight studies reported urinary levels in 423 occupationally and para-occupationally exposed subjects; 14 studies reported glyphosate levels in various biofluids on 3298 subjects from the general population. Average urinary levels in occupationally exposed subjects varied from 0.26 to 73.5 μg/L; environmental exposure urinary levels ranged from 0.16 to 7.6 μg/L. Only two studies measured temporal trends in exposure, both of which show increasing proportions of individuals with detectable levels of glyphosate in their urine over time.

**Conclusions:**

The current review highlights the paucity of data on glyphosate levels among individuals exposed occupationally, para-occupationally, or environmentally to the herbicide. As such, it is challenging to fully understand the extent of exposure overall and in vulnerable populations such as children. We recommend further work to evaluate exposure across populations and geographic regions, apportion the exposure sources (e.g., occupational, household use, food residues), and understand temporal trends.

**Electronic supplementary material:**

The online version of this article (10.1186/s12940-018-0435-5) contains supplementary material, which is available to authorized users.

## Introduction

Glyphosate, a broad-spectrum herbicide and desiccant, was first sold in 1974 and has since become the most commonly and intensively used herbicide worldwide [1]. It is available in a variety of chemical forms, such as isopropylamine salt, ammonium salt, diammonium salt, dimethylammonium salt, and potassium salt [1]. Glyphosate is mixed with other chemicals known as “inert ingredients” to constitute glyphosate based herbicides, which include the popular “Roundup®” and “RangerPro®” products that are used in agricultural fields and home gardens. The widespread application of glyphosate and GBH to crops has spurred the spread of tolerant and resistant weeds in the US, and worldwide, which in turn has created the need for more frequent applications at higher concentrations [1]. Individuals may be exposed to glyphosate through various routes such as food and drinking water, both in the occupational and environmental settings [2]. Recent findings suggest glyphosate and its metabolites may also spread by wind and water erosion [3]. Glyphosate has also been found in dust within non-agricultural homes, suggesting that the exposure is not only occupational [4]. Glyphosate levels in human beings can be quantified by measuring levels of either glyphosate or its metabolite, AMPA.

In recent years, the carcinogenic potential of glyphosate has been under review and debate by multiple authoritative and regulatory bodies. In 2015, IARC classified glyphosate as a “*probable human carcinogen*” [5], although in the same year EFSA declared that “*glyphosate is unlikely to pose a carcinogenic hazard to humans*” [6] based on typical, expected exposures to the general public. The US EPA reviewed the carcinogenic potential of glyphosate in 2016 and concluded that it is “*not likely to be carcinogenic to humans*” [7] based on typical, non-occupational exposures. The difference in conclusions are likely the consequence of some studies being excluded from the EFSA review, and some unpublished data being included in the EPA review [5–7]. The controversy over glyphosate’s carcinogenic classification is based on various aspects, including differences in the weight placed on the results of human epidemiological studies. The details of this complex debate are beyond the scope of this current review. Here, we aim to understand the current information about glyphosate exposure levels and patterns in humans.

Despite the growing and widespread use of glyphosate, evidence of bioaccumulation of glyphosate and GBH observed in rodent models [8], as well as increasing concerns for and debates about adverse health outcomes across the population, very few studies have evaluated overall human exposure. Here, we review published research documenting human exposure among workers and the general population, including changes over time, to provide crucial exposure information that could inform future risk assessments.

## Methods

We conducted a review of scientific publications on glyphosate levels in humans, including both the general population and occupationally exposed workers. PubMed and Google Scholar searches were performed using the following search terms: “glyphosate” (“glyphosate” OR “1071-83-6” OR “roundup” OR “N-(Phosphonomethyl) glycine”) or (((“AMPA”) NOT “AMPA receptor”)) OR “Aminomethylphosphonic acid”) AND (“human”). The IARC carcinogen evaluation [5] the EPA Revised Glyphosate Issue Paper [7], and several other publications were also reviewed for additional relevant articles. Finally, the references from each selected paper were manually reviewed for additional pertinent studies. No limitation on language was imposed on the search.

**Fig. 1:**
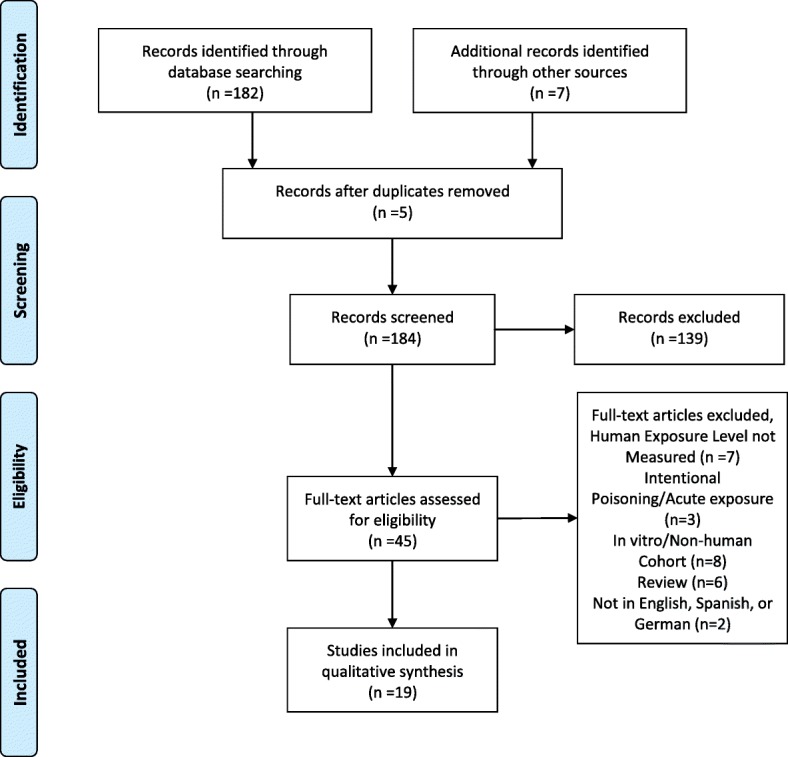
PRISMA diagram of articles included in study

The search returned a total of 189 publications, five of which were duplicates. After an abstract review, 139 studies were excluded because they were not pertinent, leaving 45 articles to review as full-text. Of these, 26 studies were excluded because they were in vitro studies, did not include data on humans, only focused on detection in the environment and not in human, or were editorials or review articles with no original data. The remaining 19 studies were used for the present review (Table 1). Five of these studies investigated occupational and para-occupational exposure to glyphosate, 11 studies documented exposure in the general population, and three reported on both (Fig. 1). Two raters reviewed the studies independently for quality based on the quality assessment tool published by the NIH [9], and discrepancies were discussed until consensus was reached. The mean quality score was 7.3 (Additional file 1: Table S1).

**Table 1:** Description of the studies included in the review

Citation number, Author, year	Country	Year of sampling	Subjects	Number of subjects	Lab methods	Type of sample	LOD glyphosate	LOD AMPA	Glyphosate Results	AMPA Results
OCCUPATIONAL EXPOSURE										
[11] Acquavella, 2004^a^	US (South Carolina, Minnesota)	NR	Farms families on application day and 3 days later	48 farmers, 48 spouses, 79 children (4–18 years old)	HPLC	Urine	1 μg/L	NR	Farmers geometric mean ± SD: 3.2 ± 6.4 μg/L (range < 1–233) on application day; 1.0 ± 3.6 (< 1–68) μg/L on day 3. Less than 25% of spouses or children had detectable values	NR
[12] Curwin, 2007	US (Iowa)	2001	Farm households	24 fathers, 24 mothers, 66 children	FCMIA	Urine	0.9 μg/L	NR	Adjusted geometric mean, farm fathers: 1.6 μg/L (1.1, 2.4); farm mothers: 1.1 μg/L (0.71, 1.8); farm children: 1.9 μg/L (1.3, 2.5)	NR
[13] Jauhiainen, 1991	Finland	1988	Forest workers sprayed a 8% Roundup containing solution for 6 h/day for 1 week	5	GC with a 63Ni-electron capture detector	Urine	100 μg/L	50 μg/L	Urine samples remained < LOD for G One urine sample further quantified had 85 μg/L glyphosate	Urine samples remained <LOD for AMPA
[15] Mesnage, 2012	France	NR	Farmer and his family, using glyphosate based herbicide	5	LC-MS	Urine	1 μg/L	NR	Concentration of 9.5 μg/L after spraying in the farmer, 2 μg/L 2 days later; 2 μg/L was also measured in one child 2 days after spraying. The mother and 2 other children had no detectable levels	NR
[14] Connolly, 2017	Ireland	2015	Amenity horticulturalists, before and after spraying	17 males, 1 female	LC MS-MS	Urine	0.5 μg/L	NR	Pre-spraying mean ± SD: 0.71 ± 0.92; post-spraying: 1.35 ± 2.18 μg/L	NR
[16] Connolly, 2018b	Ireland	2016–2017	Amenity horticulturists, before and after spraying and peak samples	18 males, 2 females	LC MS-MS	Urine	LOQ: 0.5 μg/L	NR	Pre-spraying mean (SD): 1.08 (1.20) μg/L;Post spraying: 1.72 (1.53) μg/L; Peak sample: 2.53 (1.89) μg/L	NR
[17] Rendón-von Osten, 2017	Mexico	NR	Farmers	76	ELISA	Urine	0.05 μg/L (in water)	NR	Mean ± SD in farming areas: 0.26 ± 0.23 μg/L (median: 0.28)	NR
[18] Jayasumana, 2015	Sri Lanka	NR	Healthy farmers from areas with chronic endemic kidney disease	10	ELISA	Urine	0.6 μg/L	NR	Median: 73.5 (range: 40.2- > 80) μg/L	NR
TOTAL (*n* = 7)				403						
GENERAL POPULATION										
[12] Curwin, 2007	US (Iowa)	2001	Non-farm households	23 fathers, 24 mothers, 51 children	FCMIA	Urine	0.9 μg/L	NR	Adjusted geometric mean, non farm fathers: 1.5 μg/L (1.2, 2.0); non farm mothers: 1.2 μg/L (0.91, 1.6); non-farm children: 2.5 μg/L (2.1, 3.1), range: 0.1–9.4; 65% of non farm mothers and 88% of non-farming children ≥LOD	NR
[21] McGuire, 2016^a^	US, (Washington and Idaho)	2014–2015	Lactating women> 18 years old	41 women (41 milk; 40 urine)	LC-MS	Milk, urine	Milk: 1.0 μg/L; Urine:0.02 μg/L	Milk: 1.0 μg/L; Urine:0.03 μg/L	Milk: G < LOD. Urine: G mean: 0.28 ± 0.38 μg/L, G detectable in 37/40 urine No statistically significant differences between living in urban or suburban area, or eating organic or conventional	Milk: AMPA<LOD Urine: AMPA mean: 0.30 ± 0.33 μg/L.
[22] Aris, 2011	Canada	NR	Pregnant and non pregnant women, similar in age and BMI	30 pregnant, 39 non pregnant women, 30 umbilical cords	GC-MS	Maternal and umbilical cord serum	15 μg/L	10 μg/L	G not detected in pregnant women or umbilical cord. Non pregnant women: mean 73.6 ± 28.2 μg/L.	AMPA not detected in any of the samples
[23] Parvez, 2018	US (Indiana)	2015–2016	Pregnant women age 18–39 years	71	LC-MS-MS	Urine and drinking water	Urine: 0.1 μg/LWater: 0.2 μg/L	NR	Urine: mean (SD) 3.40 (±1.24) μg/L. G not detected in drinking water	NR
[19] Connolly, 2018a	Ireland	2017	Irish adults over the age of 18 without specific dietary habits; occupation did not involve use of pesticides	50	LC-MS-MS	Urine	0.5 μg/L	NR	47 samples were tested with urinary creatinine between < 3.0 or > 30 nmol/L. 20% of samples had G levels > LOD. Median of samples with G levels above the LOD (Range): 0.87 (0.80–1.35) μg/L.	NR
[24] Knudsen, 2017	Denmark	2011–2012	Children 6–11 years and their mothers in rural and urban communities	13 mothers, 14 children	ELISA	Urine	2.5 ppb^b^	NR	Children mean: 1.96 (range: 0.85–3.31) μg/L; mothers mean: 1.28 (range: 0.49–3.22) μg/L	NR
[25] Krüger 2015^c^	Germany	2009	NR	2009	ELISA	Urine	0.0751 μg/L	NR	Mean: 1.08 μg/L^d^, maximum value: 4.2 μg/L. Highest concentration (1.55 μg/L) in 0–19 years and lowest concentration (0.77 μg/L) in > 70 years old.	NR
[26] Krüger, 2014	Germany	NR	Individuals with conventional or organic diet	99 conventional diet; 41 organic diet	GC-MS	Urine	NR	NR	Urinary level: 1.8 μg/L ^d^; subjects on conventional diet significantly higher than subjects using organic food, whose urinary values were around 0.5 μg/L^d^	NR
[27] Conrad, 2017	Germany	2001–2015	Individuals aged 20 to 29 years	399	GC-MS-MS	Urine	LOQ: 0.1 μg/L	LOQ: 0.1 μg/L	G: 127 samples (31.8%) > LOD; Males had the highest levels	AMPA: 160 (40.1%) > LOD.
[28] Hoppe, 2013	18 European countries	2013	Volunteers	182	GC-MS-MS	Urine	LOQ: 0.15 μg/L	LOQ: 0.15 μg/L	44% of samples > G LOQ; Highest G concentration: 1.8 μg/L (Latvia	36% > LOQ AMPA;) highest AMPA concentration: 2.6 μg/L (Croatia)
[17] Rendón-von Osten, 2017	Mexico	NR	Fishermen in urban area	8	ELISA	Urine	0.05 μg/L (in water)	NR	Mean ± SD in urban areas: 0.16 ± 0.1 μg/L (median: 0.20)	NR
[29] Varona, 2009	Colombia	2006	Individuals living in areas treated with aerially administered glyphosate	112	GC with electron micro-capture detector	Urine	0.5 μg/L	1.0 μg/L	G:7.6 ± 18.6 μg/L (Mean ± SD; range: 0–130 μg/L); 4/42 subjects with quantifiable G levels had quantifiable AMPA levels: mean G: 58.8 μg/L (range: 28–130 μg/L)	AMPA: 1.6 ± 8.4 μg/L (range: 0–56 μg/L)
[18] Jayasumana, 2015	Sri Lanka	NR	Healthy non-farmers from areas without chronic endemic kidney disease	10	ELISA	Urine	0.6 μg/L	NR	Median: 3.3 (1.2–5.5) μg/L	NR
[20] Kongtip, 2017	Thailand	2011	Pregnant women age 19–35 years who delivered a baby in participating hospital	82	HPLC	Maternal and umbilical cord serum	0.4 μg/L	NR	Maternal serum median: 17.5 (range 0.2–189.1) μg/L; Umbilical cord serum: 0.2 (range 0.2–94.9) μg/L 46.3% maternal serum samples < LOD, 50.7% of umbilical cord serum samples < LOD	NR
TOTAL (*n* = 14)				3298						

Note: *AMPA* aminomethylphosphonic acid, *ELISA* enzyme-linked immunosorbent assay, *FCMIA* Fluorescence covalent microbead immunoassay, *G* Glyphosate, *GC* Gas chromatography, *HPLC* High-performance liquid chromatography, *LC* Liquid chromatography, *LOD* limit of detection, *MS* mass spectrometry, *MS/ MS* tandem mass spectrometry, *NR* not reported

^a^Sponsored by Monsanto

^b^From manufacturer’s protocol (ppb = parts-per-billion, 10^− 9^)

^c^Partially overlaps with Krüger, 2014

^d^Values manually extracted from figures of the paper

We extracted data on sample size, average glyphosate concentration, laboratory technique, and population from each publication. Data were checked for accuracy by two reviewers. Units for reported averages were standardized to μg/L. Included studies reported summary estimates in a variety of ways, as arithmetic means, geometric means or medians. To display the data in the figures, we report the central tendency and range. When the GM was available, this was shown as the central tendency. When the GM was not available, but the median was, we assumed that the GM was equal to the median, since they should be approximately equal in a lognormal distribution. In some cases, we assumed the reported GM to be the LOD when at least 50% of the data were below the LOD. When arithmetic mean and standard distribution were reported, the GM was estimated from the arithmetic mean using the formula $GM=\frac{AM^2}{\sqrt{AM^2+{SD}_x^2}}$ where SD_x_ is the standard deviation of the data on the native scale and AM is the arithmetic mean of the data on the native scale, as proposed by Rappaport (Additional file 2: Table S2) [10]. Most papers reported ranges; in a few cases we estimated the 99% limits of the data assuming a lognormal distribution. Because of the small number of available studies and the wide variety of techniques used, a meta-analysis was not attempted for these studies.

## Results

**Fig. 2:**
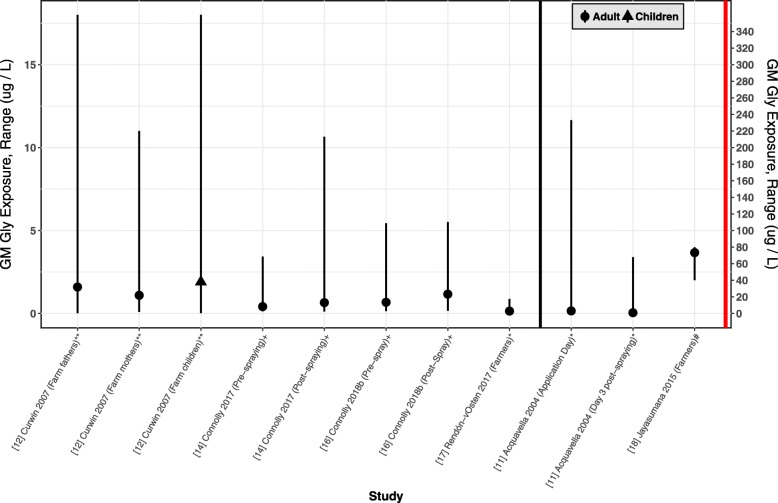
Urinary GM glyphosate concentrations in occupational and para-occupational exposure settings^&^ ^&^ Mesange 2012 excluded because values were only available from one participant. *indicates that when the lower end of the range was below the LOD, we replaced this value with 0. ** the reported range excluded values below the LOD. +values below the LOD imputed using single imputation

We reviewed eight studies that reported personal exposure to glyphosate in occupational settings; overall, 423 subjects were tested. Three of these studies reported data on para-occupational exposure and included 73 spouses and 148 children of farmworkers (Table 1). Two studies were conducted in the US [11–13], four in Europe [13–16], one in Mexico [17], and one in Sri Lanka [18]. The studies mostly involved farmers [11, 12, 15, 17]; one study recruited forest workers [13], and two focused on horticulturalists [14, 16]. The reported measures of central tendency ranged from 0.26 to 73.5 μg/L [17, 18]. All the studies involved urinary measures, although the laboratory methods and LOD varied greatly from 0.05 to 100 μg/L [13, 17]. Central tendency estimates and ranges are plotted in Fig. 2. Except for one study published in 1991 [13] including data collected in 1988, data reported in these studies was collected within the last 20 years, with the most recent sample collected in 2017.

A study conducted in South Carolina and Minnesota examined urinary glyphosate concentrations in farmers and their families (*n* = 175) the day before, day of, and 3 days after glyphosate application to crops [11]. Farm families who applied for pesticide applicators licenses were sent solicitation letters for inclusion in the study. From those willing to be contacted, farmers with families (defined as one spouse and at least one child between the ages of 4 and 18 years of age) were asked to collect the urine voids from five consecutive days and fill out pre- and post-study questionnaires detailing family activities from the week before and week of the study. Glyphosate was measured with HPLC, with an LOD of 1 μg/L. The percentage of farmers with detectable values of glyphosate was 60% on application days and declined to 27% on day three after exposure. For farmers, the GM value of glyphosate was 3.2 μg/L on the application day. The percentage of spouses with levels of glyphosate above the LOD was 2% on pre-application days, 4% on application days, and 2% on the third day post-application. The percentage of children with urinary glyphosate levels above the LOD was 7% on pre-application days, 12% on application days, and 5% by the third day post-application. The GMs were not reported for spouses or children, as they were not calculated if less than 25% of the individuals in the group had detectable values.

In a study of glyphosate concentrations among farming households in Iowa after glyphosate application conducted in 2001 (*n* = 114), the adjusted GM of glyphosate was 1.9 μg/L (95% CI: 1.3–2.5) in the urine of children in farming families (adjusted for age, sex and urinary creatinine) [12]. The fathers had a urinary creatinine adjusted GM of 1.6 μg/L (95% CI: 1.1–2.4), the mothers of 1.1 μg/L (95% CI: 0.71–1.8).

In another study, morning urine samples were collected from 76 farmers across several geographic areas in Mexico [17]. Assessment of glyphosate concentration was carried out using ELISA with a LOD of 0.05 μg/L. The mean value observed in the farming communities was 0.26 μg/L.

A small Finnish study conducted in 1988 examined five forest workers who sprayed a solution containing 8% Roundup with a brush saw for 6 hours per day [13]. Workers used limited personal protective equipment, wearing only cotton overalls, cotton or rubber gloves, hats or safety helmets and rubber boots. Rain clothes were also worn on days with precipitation. The hypothesized route of exposure was reported by the authors as skin contamination, likely due to the limited personal protective equipment and Roundup dispersed through the air. Air samples collected at midweek during spraying contained < 1.25 μg glyphosate/ m^3^ air. After a 3-week work period, the glyphosate concentration in the urine remained below detection level (< 100 μg/L). Only one urine sample was further quantified and found to contain a glyphosate concentration of 85 μg/L.

A case study in France tested the presence of glyphosate in the urine of a farmer and his family (*n* = 5) because of the occurrence of birth defects in the family [15]. Glyphosate concentration in the farmer’s urine reached a peak of 9.5 μg/L 7 h after spraying, without personal protective equipment, and plateaued at 2 μg/L 2 days after spraying. The concentration of 2 μg/L was also measured in one child 2 days after spraying. The mother and 2 other children had no detectable levels of glyphosate.

A study conducted in 2015 of amenity horticulturalists (*n* = 18) was conducted in Ireland with the aim of measuring urinary biomarkers of occupational exposures, including to glyphosate [14]. Public workers at parks and other green spaces in Ireland were asked to collect urine immediately before and after spraying glyphosate, and biosamples were analyzed with mass spectrometry (LOD: 0.5 μg/L). Pre-spraying samples had significantly lower concentrations of urinary pesticide concentrations, including glyphosate (mean: 0.71 (SD: 0.92) μg/L) compared to post-spraying samples (mean: 1.35 (SD: 2.18) μg/L).

In a similar study conducted in 2016 and 2017 on a separate population of amenity horticulturalists (*n* = 20), urinary biomarkers of glyphosate exposure were measured before, immediately after (within 1 hour), and the first urine void the morning after spraying with Roundup® at work [16]. Each worker was also given the option to collect additional urine voids. For each worker, a peak urinary glyphosate level was identified. In the study, 27% of the samples were below the LOQ, 76% of which were either pre-task samples or morning-after samples. Of the post-work samples, only 7% were below the LOQ. There was a statistically significant difference between the pre-task samples levels (mean (SD): 1.08 (1.20) μg/L) and the post-task sample levels (mean (SD):1.72(1.53) μg/L) or peak sample levels (mean (SD):2.53 (1.89) μg/L). There was not a statistically significant difference between the pre-sample levels and first morning void levels (mean (SD): 1.32 (1.32)).

In a study of 20 paddy farmers in Sri Lanka, researchers examined the urinary metabolites of pesticides, including glyphosate, and sampled well water from active and abandoned wells near the farmers to examine whether pesticides were related to kidney disease [18]. The study included 10 healthy farmers without kidney disease living in a region with endemic CKDu; their median urinary glyphosate levels was 73.5 (range: 40.2-80.0) μg/L.

**Fig. 3:**
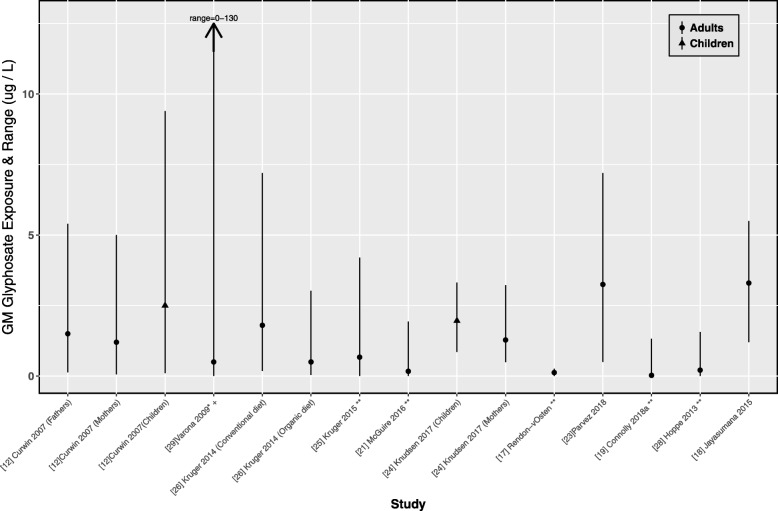
Urinary GM glyphosate concentrations in the general population. * indicates that > 50% of the values were below the LOD, and therefore the LOD was selected as the central tendency. **indicates that when the lower end of the range was below the LOD, we replaced this value with 0. +Highest value reported was 130 μg/L

We identified 14 studies reporting on glyphosate levels in biofluids from the general population, with 3298 subjects tested (Table 1). Exposure assessment in these studies was primarily based on urine samples (*n* = 11), though some studies utilized maternal milk and urine (*n* = 1) or the serum of umbilical cord and maternal blood (*n* = 2). Four studies were conducted on pregnant women. While most studies reported arithmetic means, others reported GM [12], or medians [18–20]. The arithmetic mean levels of glyphosate detected in urine samples ranged from 0.16 to 7.6 μg/L. The central tendencies and ranges of these urinary levels are presented in Fig. 3. Where possible, the GM and range are reported, or estimated from the median or arithmetic mean and reported. There was a large degree of variability in the LOD, which ranged from 0.02 to 15 μg/L [21, 22].

In a study completed in 2001 comparing farming and non-farming households in Iowa (*n* = 98) [12], glyphosate concentrations in urine of children from non-farming families ranged from 0.10–9.4 μg/L and the adjusted GM of glyphosate was 2.5 μg/L (95% CI: 2.1–3.1) (adjusted for age, sex and urinary creatinine). The fathers in these families had a urinary creatinine adjusted GM of 1.5 μg/L (95% CI: 1.2–2.0), the mothers of 1.2 μg/L (95% CI: 0.91–1.6); 65% of non-farming mothers and 88% of non-farming children had detectable levels of glyphosate in their urine.

A study conducted in 2014 and 2015 used HPLC and mass spectrometry to examine milk and urine samples from 41 lactating women in Idaho and Washington State to determine whether glyphosate and AMPA could be detected in either fluid [21]. Researchers sampled human milk and urine from women of 18 years and older who were 1–3 months postpartum and were breastfeeding and/or pumping milk at least five times per day. The LOD and LOQ for glyphosate in milk were 1.0 μg/L and 10.0 μg/L respectively, in urine were 0.02 μg/L and 0.10 μg/L respectively. The LOD and LOQ for AMPA in milk were 1.0 μg/L and 10.0 μg/L, respectively, in urine were 0.03 μg/L and 0.10 μg/L respectively. All milk samples had glyphosate and AMPA levels below the LOD. The mean ± SD urinary glyphosate level was 0.28 ± 0.38 μg/L, while the mean urinary AMPA level was 0.30 ± 0.33 μg/L. Glyphosate was detected in 37 of the 40 urine samples tested; the highest value was 1.93 μg/L. There was no statistically significant difference between glyphosate or AMPA levels in those living near an urban versus suburban area, or between self-reported diet containing mostly organic versus conventional foods.

An analogous study was conducted in Quebec, Canada on serum from 30 pregnant and 39 non-pregnant women with similar age and BMI [22]. Glyphosate assessment was conducted with mass spectrometry, with an LOD of 15 μg/L. For pregnant women, the umbilical cord was also available for analysis. Glyphosate was not detected in serum of pregnant women or in the umbilical cord. Non-pregnant women had a glyphosate mean level of 73.6 ± 28.2 μg/L. AMPA was not detected in any of the samples tested.

A study conducted in central Indiana enrolled 71 pregnant women aged 18 to 39 years during their prenatal visits in 2015 and 2016 [23]. Each participant answered an online questionnaire about their diet and demographic information and provided two urine samples during their clinical visits between 11 and 38 weeks of gestation. Participants also provided a water sample from their residential source, either public supply or private well, at the time of the second prenatal urine sample. Glyphosate levels were measured by LC-MS/MS, with a LOD of 0.2 μg/L and 0.1 μg/L in drinking water and urine, respectively. Glyphosate was detected in 93% of the urine samples, with a mean (SD) of 3.40 (1.24) μg/L. Women in rural areas had higher levels of glyphosate (mean: 4.19 μg/L, SD: 1.58 μg/L) compared to women in suburban areas (mean: 3.17 μg/L, SD: 1.13 μg/L) and urban areas (mean: 3.47, SD: 0.50 μg/L). Drinking water samples had no detectable glyphosate, which suggests that it was not a relevant source of exposure for the cohort under study.

Researchers in Mexico conducted a cohort study comparing urine glyphosate levels in farm workers with eight fishermen who lived in urban areas [17]. ELISA with a 0.05 μg/L LOD in water was used; the mean urinary glyphosate level in the urban fisherman, which could be considered a control sample of subjects not exposed through occupation, was 0.16 μg/L.

In a pilot study conducted in 2017 in Ireland, 50 adults without a specific diet who did not use pesticides as part of their profession provided first morning void urine samples for glyphosate analysis [19]. Only urine samples with creatinine levels between 3.0 and 30 nmol/L were assumed to be valid (*n* = 47). Of these samples, 10 had glyphosate concentrations above the LOD. The median concentration of glyphosate for those 10 samples was 0.87 μg/L, with a minimum value of 0.80 μg/L and a maximum value of 1.35 μg/L. Six of the 10 samples with detectable glyphosate were from women, and three were from individuals who indicated past use of glyphosate in their homes, but not within the last month. None of the three samples that were excluded due to creatinine levels had detectable glyphosate.

In a study of mothers (*n* = 13) and children (*n* = 14) conducted in 2011 and 2012 in Denmark [24], urine spot samples revealed concentrations of glyphosate above the LOD (2.5 μg/L) in both urban and rural dwelling populations. Children had higher concentrations of glyphosate in their urine than their mothers, with a mean of 1.96 (range: 0.85–3.31) μg/L compared to 1.28 (range: 0.49–3.22) μg/L in the mothers. The authors did not detect a statistically significant difference in concentrations between rural and urban populations.

Similar results showing children having higher concentrations of glyphosate than their mothers were found in a German study conducted in 2009 including 2009 volunteers [25]. The mean value for all samples was 1.08 μg/L and the maximum value 4.2 μg/L. Participants between 0 to 19 years of age had the highest mean concentrations of urinary glyphosate (1.55 μg/L); the mean concentration decreased with age and was the lowest for participants older than 70 years (0.77 μg/L).

A previous study from the same German group tested 140 urine samples from subjects with mass spectrometry and reported an average value in all subjects of approximately 1.8 μg/L [26]. A subset of 41 subjects who self-reported eating organic food had mean urinary values of approximately 0.5 μg/L, which was significantly lower than those on a conventional, non-organic diet.

Conrad et al. [27] used 24-h urine samples from 399 subjects stored in the German Environmental Specimen Bank. Samples from 20 males and 20 females aged between 20 and 29 years were collected between March and April in selected years between 2001 and 2011 and every year from 2012 to 2015; 127 samples (31.8%) contained glyphosate concentrations at or above the LOD (0.1 μg/L). The maximum glyphosate levels peaked in the years 2013 (2.80 μg/L) and 2014 (1.78 μg/L). Males had the highest median level (0.18 μg/L) in 2013. A sub-analysis of subjects who self-reported being vegetarians showed no differences compared to the values obtained from the main sample population. A more in depth discussion of the exposure trends seen in this study follows below.

A non-peer reviewed report on glyphosate residues in 182 urine samples from 18 different European countries, commissioned by the European Community in 2013, documented exposure to glyphosate and AMPA with mass spectrometry (LOQ: 0.15 μg/L) [28]. Glyphosate and AMPA were detected in 44 and 36% of the urine samples analyzed, respectively.

A study conducted in 2006 of 112 residents of several Colombian regions where glyphosate is aerially administered to eradicate illicit crops reported a mean urinary concentration of glyphosate (LOD: 0.5 μg/L) of 7.6 μg/L (SD: 18.6; range: 0 to 130 μg/L) and a mean AMPA (LOD: 1 μg/L) concentration of 1.6 μg/L (SD: 8.4; range: 0 to 56 μg/L) [29]. Of the 42 subjects with quantifiable levels of glyphosate, four had quantifiable levels of AMPA as well. In these four individuals, the mean glyphosate level was 58.8 μg/L (range: 28–130 μg/L).

A Sri Lankan study examined urinary glyphosate levels in 10 healthy non-farmers living in areas where CKDu was not endemic [18]; the mean level of glyphosate was 3.3 μg/L.

A study conducted in Thailand in 2011 recruited 82 women between the ages of 19–35 years during their seventh month of pregnancy [20]. The women were interviewed about their diet, general health, and work exposures, including potential agricultural exposures, through several questionnaires at the time of recruitment. Maternal blood serum and umbilical cord were collected and tested for glyphosate (LOD: 0.4 μg/L) after giving birth. Of the maternal serum samples, 53.7% were at or above the LOD, while 49.3% of the umbilical cord samples were at or above LOD; 30.5% of the maternal samples had levels of glyphosate between 1 and 50 μg/L, 12.2% between 51 and 100 μg/L, 7.3% between 101 and 50 μg/L and 3.7% between 151 and 200 μg/L. The median glyphosate in maternal serum was 17.5 (range 0.2–189.1) μg/L. For the umbilical cords (*n* = 75), 28.3% of the samples had levels of glyphosate between 1 and 25 μg/L, 12.0% between 26 and 50 μg/L, 5.3% between 51 and 75 μg/L and 2.7% between 76 and 100 μg/L. The median glyphosate level was 0.2 (range 0.2–94.9) μg/L. Paired comparison between maternal blood serum and cord blood (*n* = 36) indicated that maternal serum samples exhibited higher levels of glyphosate. Occupational and lifestyle factors were found to be predictive of glyphosate at or above the LOD. The odds of having detectable levels of glyphosate in blood were 11.9 (CI: 3.6–39.5) times higher for women who worked in the fields compared to those who did not. After adjusting for maternal occupation, women who lived near agricultural areas (< 0.5 km) also had higher odds of glyphosate at or above the LOD (OR: 4.2, CI: 1.4–12.3) than those who lived further away.

There is limited information regarding secular trends in glyphosate exposure. In 2017, Mills et al. reported the excretion of glyphosate and AMPA in participants from the Rancho Bernardo Study of Healthy Aging, a study that began in 1972 by monitoring 6629 adults greater than 50 years of age who were residing in Southern California [30]. A small subset of this population (*n* = 112) had routine morning spot urinary biospecimens taken at all five clinic visits from 1993 to 2016; 100 of these 112 individuals were randomly chosen for urinary measurements of glyphosate and AMPA using chromatography and mass spectrometry. The LODs were 0.03 μg/L for glyphosate and 0.04 μg/L for AMPA. Urinary concentrations were normalized to each sample’s specific gravity to account for dilution. The mean glyphosate concentrations were 0.02 (95% CI: 0.01–0.04) μg/L in samples taken between 1993 and 1996, and 0.31 (95% CI: 0.24–0.39) μg/L in samples taken between 2014 and 2016. The percentage of participants with glyphosate above the LOD increased from 12% for the period 1993–1996 to 70% for the period 2014–2016. The mean levels of AMPA were 0.01 (95% CI: 0.00–0. 02) μg/L between 1993 and 1996, and 0.29 (0.217–0.35) μg/L between 2014 and 2016. During the same period, the percentage of participants with AMPA levels above the LOD increased from 5 to 71%.

The previously mentioned study by Conrad et al. conducted in Germany used 24-h urine samples from 399 subjects stored in the German Environmental Specimen Bank and examined time trends in exposure [27]. The LOQ for glyphosate was 0.1 μg/L. across all 14 years; 31.8% of the samples tested had glyphosate concentrations and 40.1% had AMPA concentrations at or above the LOQ. The percentage of individuals with glyphosate levels higher than the LOQ was 10% in 2001 and showed the highest percentages in 2012 (57.5%) and 2013 (56.4%). The maximum concentrations of glyphosate measured in urine peaked in 2013, with 2.80 μg/L for men and 1.78 μg/L for women. Values plateaued in the following 2 years.

## Discussion

The current review, covering 19 studies deemed suitable for inclusion, highlights the paucity of data and associated data gaps on internal glyphosate levels among individuals exposed occupationally, para-occupationally, or environmentally to the herbicide. As such, it is challenging to fully understand the extent of exposure among workers or the general population. The situation is compounded by the fact that these few available studies utilize different methodologies, measurements, and approaches to reporting their results, making it difficult to distill the evidence of exposure to glyphosate across studies.

More specifically, we observed several crucial data gaps in the literature we reviewed on potential occupational exposure to glyphosate: very few studies specifically assessed occupational exposure before and after using glyphosate-based products; only one study measured urine samples before and after spraying in a very small sample of 18 amenity horticulturalists workers, while two studies only measured during spraying or after spraying. Furthermore, no study was designed to tackle the hypothesis of seasonality in exposure, including changes associated with the time of the year that the crop is harvested, the type of crop, and the location of the farm in one or the other hemisphere. Additionally, most of the studies have been conducted in the US and Europe, using small samples of farmers and collecting a one-time spot urine; consequently, generalizability is limited. The limited data on occupational exposure is particularly concerning given the magnitude and frequency of glyphosate use in agriculture worldwide [1]. Additionally, to our knowledge, there is a complete lack of data on glyphosate exposure among workers involved in the manufacturing and processing of glyphosate and GBHs, which is highly concerning given their potential toxicities [5, 31].

Among the general population, the current information available suggests that mean levels of glyphosate in urine samples are generally below 4 μg/L [12, 21, 23, 24, 26]. However, in areas where aerial spraying is administered, mean urinary concentrations in the population above the LOD can reach as high as 7.6 μg/L [29]. As with the literature on occupational exposure, studies of environmental exposure have significant gaps: most of the residential exposure studies have been conducted in US and Europe. There are also limited data on geographic variability in exposure levels across the general population. Only one study reported on urinary levels in South America [29], despite the fact that glyphosate is widely used and sprayed all over the continent as part of the anti-recreational drug strategy [31]. The similarity between average levels of glyphosate measured in the general population and the occupationally exposed is an unusual finding. It suggests that there are unmeasured, inevitable high-exposure episodes occurring during daily life activities, not addressed by any regulatory assessment anywhere in the world. This gap in data and risk assessment renders current regulatory appraisals largely irrelevant to those who experience these unusual, high-end exposures. Studies like Kongtip et al. [20] show that even expectant mothers, a population that typically avoids excess chemical exposure, can have serum glyphosate levels as high as 189 μg/L.

The few studies that report exposure among both children and adults indicate that children exhibit higher levels of glyphosate in biofluids than adults [13, 21, 32]. The reasons for this distinction are not clear but could be due to higher relative intake of contaminated food and water, differences in metabolism and elimination, and/or differences in behavior and activity patterns. These findings require further investigation, given the particular vulnerability of children to chemical exposures [33].

There are also some overarching methodological aspects that need comment. Available studies were conducted with different laboratory methodologies, primarily LC and GC mass spectrometry and ELISA. Research presented at the Asia Pacific Association of Medical Toxicology conference suggests that LC-MS may be more sensitive than GC-MS or ELISA at detecting glyphosate in urine samples, creating an additional source of variation [34]. Additionally, LODs and LOQs vary greatly across studies and over time. Variation in LOQs impacts calculation of average levels, and it also prevents the integration of data across studies and over time and an understanding of the impacts of how changing LOQs affect “average” residue levels. Finally, only seven studies adjusted for creatinine the average glyphosate level reported [11, 12, 14, 16, 18, 19, 27]. Kidney disease, reflected by creatinine levels, may affect the excretion of pesticides including glyphosate [18], further adding to the potential for variation in the data. Despite these limitations, we made an effort to standardize the data where possible so that regional and temporal exposure variations could be seen. When ranges are calculated or reported it appears evident that some subjects present very high levels of urinary glyphosate, and that overall there is a large variability in individual levels. This may be a reflection of differences in daily exposure, or in the metabolic ability to tackle the chemical once it is in the body.

This review serves to highlight future research directions in this field: additional studies involving larger segments of the population, including in diverse geographic areas, apportioning the exposure sources (e.g., occupational, household use, food and drink residues) are needed in order to improve the knowledge of the extent of glyphosate exposure. It is surprising that the NHANES, a federally funded program that has assessed the health and nutritional status of adults and children in the US since 1959, has not monitored urinary and plasma glyphosate or AMPA levels in biofluid samples [35], despite the fact that it reports on several other pesticides, including other organophosphates. Adding glyphosate and AMPA to NHANES would also address another aspect noted in this review, namely the variability in the type of specimen utilized (urine, serum, umbilical cord, maternal milk) across studies. Monitoring both glyphosate and AMPA levels would provide a more robust picture of their relationship, as AMPA and glyphosate levels do not correlate well, likely due to individual genetic differences in metabolism capacity, or to exposure to other chemicals which can degrade into AMPA [28]. Monitoring inert ingredients present in GBHs may help to illuminate any interaction between these components and glyphosate. National biomonitoring would cover diverse segments of the population with an adequate sample size and include common biological fluids and laboratory methods; thus, we strongly suggest the inclusion of glyphosate in upcoming NHANES assessments.

The present review documents that there is limited information available about glyphosate levels in the general population, despite the fact that glyphosate is detected in dust, food and water. For example, Curwin et al. detected glyphosate in the dust of both farming and non-farming households, indicating that this exposure extends beyond occupational settings [4]. The EPA completed a glyphosate food risk assessment 10 years ago and evaluated the levels of pesticide residues in food, drinking water, grain based beverages, and residues encountered through non-occupational sources such as in homes, recreational areas, and schools using the National Health and Nutrition Examination Survey/ What We Eat In America from 2003 to 2008 [2]. The residues in food ranged from 100 μg/L in vegetables such as tomatoes and pepper to 200,000 μg/L in peppermint and peppermint oils. However, given the increasing rates of glyphosate usage over the past decade, it is likely that this EPA assessment does not reflect current potential exposure sources and levels. Several European studies have also examined the level of glyphosate found in foods, including produce and grains for human consumption as well as feed for chickens. These studies report measurable levels in many food products [7, 26, 36, 37], including the muscle and organ tissues of chickens and cows [35, 36]. An FDA review of glyphosate levels in food in the United States found that over 60% of corn and soybean samples analyized had detectable glyphosate residues, and the Environmental Working Group sampled 28 kids’ cereal products and found detectable levels of glyphosate in all of them and levels of glyphosate exceeding 160 μg/L in 26 of them [38, 39]. Glyphosate and AMPA have also been detected in water. In the EPA’s *Dietary Exposure Analysis in Support of Registration*, which utilized monitoring data from the USGS, the agency estimated the worst-case scenario for a chronic dietary assessment as 75 μg/L in water [2]; similar results have been observed in studies conducted in Europe [40].

From our review, it also appears that there is limited information on the temporal change in glyphosate levels in the general population and in occupational settings, even though usage of GBHs has increased greatly in recent years [1]. The two available studies reporting repeated measurements during the late 1990s through 2016 were conducted in only two geographic regions on a very small sample size (100 subjects in California, 399 subjects in Germany), and while strongly suggestive that there may be an upward trend in population average exposure over time, as well as a large variability in individual levels, are hardly generalizable to the general public because of unknown variation across study populations, timing of outcome measure collection, and proximity to areas sprayed with GBHs.

## Conclusion

In summary, additional studies are urgently needed to evaluate levels of glyphosate and related metabolites in the general population and in workers, including across different geographic areas, apportioning the exposure sources and considering changes in these measures over time. Improved exposure assessment is necessary for conducting accurate risk assessments and high quality epidemiological studies. This work is crucial given the substantial increase in glyphosate use in recent years [1] and the current questions of carcinogenicity under debate by health and environmental agencies around the world [5].

## Additional files

Additional file 1: **Table S1.** Quality Rating by Article. (DOCX 17 kb)

Additional file 2: **Table S2.** standardized values of glyphosate used for figures – geometric means and ranges. (DOCX 23 kb)

## Abbreviations

AMPA: Aminomethylphosphonic Acid
BMI: Body Mass Index
CKDu: Chronic Kidney Disease of Unknown Origin
EFSA: European Food Safety Authority
ELISA: Enzyme-Linked Immunosorbent Assay
EPA: Environmental Protection Agency
GBH: Glyphosate-based herbicides
GM: Geometric Mean
HPLC: High-Performance Liquid Chromatography
IARC: International Agency for Research on Cancer
LC-MS/MS: Liquid Chromatography- Tandem Mass Spectrometry
LOD: Limits of Detection
LOQ: Level of Quantification
NHANES: National Health Nutrition and Examination Survey
NIH: National Institutes of Health
USGS: United States Geological Survey
