# Supplementary material for: The evidence of human exposure to glyphosate: a review
Source: Environ Health. 2019 Jan 7;18:2. doi: 10.1186/s12940-018-0435-5 (PMC6322310; doi:10.1186/s12940-018-0435-5)
Supplement: Supplementary file 2 — Table S2. standardized values of glyphosate used for figures – geometric means and ranges. (DOCX 23 kb) [file 12940_2018_435_MOESM2_ESM.docx]

| **Author, year** | **Number of subjects/biospecimens** | **Transformed Glyphosate Results (ug/L):**  **Central tendency and range** | |
| --- | --- | --- | --- |
| **OCCUPATIONAL EXPOSURE** |  |  | |
| Acquavella, 2004^a^ | 48 farmers | Application day:  GM: 3.2  Range: 0-233^1^  % > LOD: 60%  Day 3 post-spraying:  GM: 1.0  Range: 0-68^1^  % > LOD:27% | |
|  | 48 spouses | Application day:  GM: not calculated (<25% had detectable values)  Range: 0-2^1^  % > LOD: 4%  Day 3 post-spraying:  GM: not calculated (<25% had detectable values)  Range: 0-1^1^  % > LOD: 2% | |
|  | 79 children | Application Day:  GM: not calculated (<25% had detectable values)  Range: 0-29^1^  % > LOD: 12%  Day 3 post-spraying:  GM: not calculated (<25% had detectable values)  Range: 0-6^1^  % > LOD: 5% | |
| Curwin, 2007 | 24 fathers | GM: 1.6  Range: 0.02- 18^2^  %> LOD: 75% | |
|  | 24 spouses | GM: 1.1  Range: 0.10-11^2^  %> LOD: 67% | |
|  | 65 children | GM: 1.9  Range: 0.22-18^2^  %> LOD: 81% | |
| Jauhiainen, 1991 | 5 | Urine samples remained < LOD  One urine sample further quantified had 85 µg/L glyphosate | |
| Mesnage, 2012 | 1 farmer | Post-spraying: 9.5 μg/L  2 days post-spraying: 2 | |
|  | 1 spouse | <LOD | |
|  | 3 children | 2 Days post-spraying  1 child: 2  2 other children: <LOD | |
| Connolly, 2017 | 17 | Pre-spraying  GM: 0.42  Range: 0.13-3.43  % > LOD: 35%    Post-spraying  GM: 0.66  Range: 0.12-10.66  %> LOD: 55% | |
| Connolly, 2018 | 20 | Pre-spraying  GM: 0.68  Range: 0.14-5.44  %>LOD: 62%  Post-spraying  GM: 1.17  Range: 0.17-5.51  % > LOD: 82% | |
| General Population |  |  | |
| Curwin, 2007 | 23 fathers, | | GM: 1.5  Range: 0-5.4^1^  %>LOD: 66% |
|  | 24 spouses | | GM: 1.2  Range: 0-5.0^1^  %>LOD: 65% |
|  | 51 children | | GM: 2.5  Range: 0-9.4^1^  %>LOD:88% |
| McGuire, 2016^a^ | 41 women (41 milk; 40 urine) | | Milk: <LOD.  Urine:  GM: 0.17^4^  Range: 0- 1.93  %>LOD: 92.5% |
| Aris, 2011 | 30 pregnant women (serum & umbilical cords) | | Serum: <LOD  Umbilical cord: <LOD |
|  | 39 nonpregnant women | | AM: 15^8^  Range: 0-93.6^1^  %>LOD: 5% |
| Parvez, 2018 | 71 | | Urine  GM: 3.25^3^  Range: 0.5-7.2  Drinking water: <LOD |
| Connolly, 2018 | 50 | | GM: 0.025^3^  Range: 0- 1.32^1^  %>LOD: 20% |
| Knudsen, 2017 | 13 mothers | | AM: 1.28  Range: 0.49-3.22  %>LOD: 100% |
|  | 14 children | | AM: 1.96  Range: 0.85-3.31  % > LOD: 100% |
| Krüger 2015^c^ | 2009 | | GM: 0.67^3,5^  Range: 0-4.2^1^  %>LOD: 99.6% |
| Krüger, 2014 | 99 conventional diet | | AM: 1.8^6^  Range: 0.18-7.2^6^  %>LOD: NR |
|  | 41 organic diet | | AM: 0.5^6^  Range: 0.04-3.02^6^  %>LOD: NR |
| Conrad, 2017 | 399 | | 2001:  GM: 0.1^3,8^  Range: 0-0.40^1^  %>LOD: 10%  2003:  GM: 0.1^3,8^  Range: 0-0.37^1^  %>LOD: 17.5%  2005:  GM: 0.1^3,8^  Range: 0-0.26^1^  %>LOD: 30%  2007:  GM: 0.1^3,8^  Range: 0-0.26^1^  %>LOD: 15%  2009:  GM: 0.1^3,8^  Range: 0-0.30^1^  %>LOD: 27.5%  2011:  GM: 0.1^3,8^  Range: 0-0.51^1^  %>LOD:32.5%  2012:  GM: 0.11^3^  Range: 0-0.63^1^  %>LOD: 57.5%  2013:  GM: 0.11^3^  Range: 0-2.8^1^  %>LOD: 56.4%  2014:  GM: 0.1^3,8^  Range: 0-1.78^1^  %>LOD:32.5%  2015:  GM: 0.1^3,8^  Range: 0-0.57^1^  %>LOD 40% |
| Hoppe, 2013 | 182 | | AM: 0.21  Range: 0-1.56^1^  %>LOQ: 44% |
| Rendón-von Osten, 2017 | 8 | | GM: 0.12^7^  Range: 0-0.26^1^ |
| Varona, 2009 | 112 | | AM: 0.5^8^  Range: 0-130^1^  %>LOD: 39.6% |
| Jayasumana, 2015 | 10 | | GM: 3.3^3^  Range: 1.2-5.5  %>LOD: NR |
| Kongtip, 2017 | 82 serum samples | | GM: 17.5^3^  Range: 0-189.1^1^  %>LOD: 53.7 |
|  | 75 umbilical cord samples | | GM: 0.4^8^  Range: 0-94.9^1^  %>LOD: 49.3% |
| **TOTAL (n= 14)** | **3298** | |  |

^1^ indicates that when the lower end of the range was below the LOD, we replaced this value with 0

^2^ reported range excluded individuals below the LOD

^3^ GM estimated from median

^4^ GM estimated from AM

^5^ median estimated from table

^6^ AM & range estimated from plot

^7^ GM calculated from supplemental data

^8^ >50% of the data was below the LOD, thus the LOD was used as the central tendency
